# Impacts of climate and human activities on Daihai Lake in a typical semi-arid watershed, Northern China

**DOI:** 10.1371/journal.pone.0266049

**Published:** 2022-05-24

**Authors:** Yajun Du, Weifeng Wan, Qingbo Li, Haifeng Zhang, Hui Qian, Jinlong Cai, Junzhi Wang, Xiaokang Zheng

**Affiliations:** 1 Yellow River Engineering Consulting Co., Ltd. (YREC), Zhengzhou, Henan, China; 2 Key Laboratory of Water Management and Water Security for Yellow River Basin, Ministry of Water Resources (under construction), Basin, China; 3 School of water and Environment, Chang’an University, Xi’an, Shaanxi, China; Universiti Sains Malaysia, MALAYSIA

## Abstract

A rapid shrinkage of Daihai Lake was found in recent decades. The present study analyzed the characteristics of Daihai Lake shrinkage and quantified the contribution of climate and human activities. The results of Mann-Kendall- Sneyers test and moving t-test showed that there was an obvious mutation point of lake level in 2006 and the descending speed of Daihai Lake level post-2006 (-0.46m/a) was 3.22 times that of pre-2006 (-0.14m/a). The centroid of Daihai Lake moved 1365.18 m from southwest to northeast during 1989 ~ 2018 with an average speed of 47.08 m/a. The results of Mann-Kendall trend test revealed that the annual evaporation showed a significant downward trend with a rate of approximately -5.33 mm/a, while no significant trend was found in precipitation. Daihai lake water level showed a very weak relationship with evaporation (r = 0.078, p < 0.01) and precipitation (p>0.05) respectively. Daihai Lake was influenced by human activities mainly from land use/ land cover, building reservoirs, pumping groundwater and directly consuming Daihai Lake water by Daihai power plant (DHPP). It was thought-provoking that DHPP began to consume Daihai lake water in 2006, which was consistent with abrupt change of Daihai lake level. The proportion of human impact was fluctuating upward. Human factors were the main factor of lake water reduction in last 10 years and the 5-year average contribution of human activities to Daihai Lake shrinkage was more than 61.99%. More attention and economic support should be given to prevent the continuous shrinkage of Daihai Lake.

## Introduction

Lakes are key components of the hydrological cycle and provide many important benefits to society including drinking water, flood attenuation, nutrition, and recreation [1]. As lakes in semiarid regions are inland water bodies recharged mainly by local water sources, lake water area reflects the spatial and temporal patterns of change in regional climate and human water consumption [2, 3]. By nature, the water level in lakes, and endorheic lakes in particular, is a sensitive sentinel of changes in hydrologic balance [1].

Lake shrinkage is an issue of increasing concern in dryland regions [2, 4]. Lake Chad in Africa decreased by about 40% in area from the 1960s to the year 2000 [2]. Lake Aksehir in Turkey decreased from 342.9 km^2^ in 1975 to 84.9 km^2^ in 2006 and became desiccated in 2008 [5]. At a regional scale, 5−9% (by number) or 11−13% (by area) of lakes in southern Siberia vanished between the 1970s and the end of the 1990s [6]. Many lakes of great ecological, historical, and cultural values located in arid/semi-arid area of northern China are under threat of vanishing, where surface inflow to the lakes has decreased significantly [7]. There were 121 of the 241 lakes in the semi-arid region of China bordered to the Asian Gobi desert became fully desiccated at the end of the 2000s [2]. Tao, et al. [8] found a rapid loss of lakes on Mongolian Plateau in the past decades and the number of lakes with a water surface area larger than 1 km^2^ decreased from 785 in the late 1980s to 577 in 2010. Compared with 2000, the total area of lakes larger than 0.5 km^2^ in Inner Mongolia has decreased by 1315.23 km^2^ by around 2010 [9]. Due to climate change and increasing exploitation of groundwater resources, this downward trend of lakes will continue in the following decades in arid/semi-arid regions [10].

The impacts of climatic variation and human activities are considered as the most important factors for the recent lake changes [3]. Due to population growth and the accompanying development of industry and agriculture, human activities affect lakes by intensified exploitation of fisheries resources, reclamation of land from marshes of lakes, wastewater discharge, construction of water conservancy, and tourism. The environmental changes of lakes can greatly affect the available freshwater resources, lead to the consequential evolution of regional ecological environment, and have a critical influence on regional sustainable development [11]. Climate change and human activities pose great threats to lake systems which plays a crucial role in supporting the natural environment and livelihoods [3], while the process and major driving factors of the change of lake water level in different regions are different.

Daihai Lake is located in a semi-arid area of Northern China, where annual evaporation far exceeds precipitation. In recent years, the water level of Daihai Lake declined rapidly and the ecological environment deteriorated seriously. As a typical terminal lake, Daihai Lake is typically sensitive to the effects of local evaporation and replenishment activities. Determination of causes and consequences of the changes of lakes is the precondition of solutions for their protection and restoration [11], while the main reasons for the shrinkage of Daihai Lake water have been controversial in recent decades. Sun [12, 13] reported that warmer climate and less precipitation lead to a dry tendency under the global climate changes and human activities in the Daihai wetland in the past 50 years, and the decline in the Daihai Lake level over the last 20 years of the twentieth century had been mainly due to the intensification of human activities in the watershed. Cao [14] reported that the main reason for the water level change in Daihai Lake in the late last century was climate change, because the partial correlation coefficient between the changes in the annual water level of Dai Lake and the annual precipitation during 1960~1996 was relative larger (0.76). These studies mainly focus on the qualitative analysis of lake shrinkage and the contribution of climate change and human activities on Daihai Lake shrinkage is not clear. Additionally, the abrupt changes of Daihai Lake water were less studied by previous research [12, 15, 16].

The present study aimed to advance the understanding of the key processes controlling the lake water level variation over the last three decades. This paper intends to take Daihai Lake as the research object to quantitatively analyze the water balance of Daihai Lake, so as to reveal the changes of recharge into the lake and the reasons for the shrinkage of Daihai Lake.

## Materials and methods

### Geography and geology

**Fig 1:**
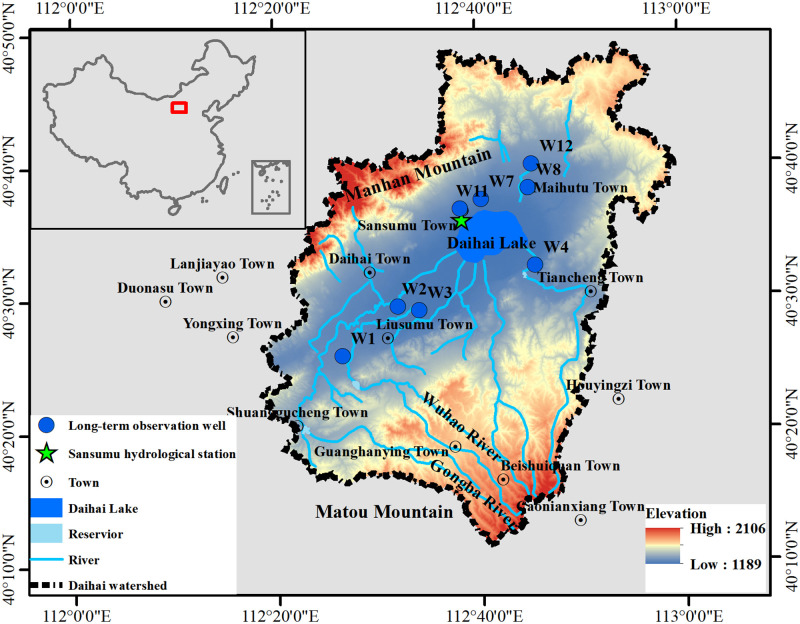
Geographical location of Daihai Lake and Daihai watershed (the map was prepared in ArcGIS 10.2 using political boundaries from the National Geomatics Center of China (http://www.ngcc.cn/ngcc/) and digital elevation model from the U.S. Geological Survey (https://www.usgs.gov/), “Esri reserves the right to grant permission for any other use of the image”).

Daihai Lake (40°28’~ 40°37’ N, 112° 33’ ~ 112°47’ E) administratively belongs to Liangcheng County, Ulanqab city, Inner Mongolia Autonomous Region. It is a typical inland lake in semi-arid area and is the third-largest inland lake in Inner Mongolia following after Hulun Lake and Dalinuoer Lake. Daihai Lake locates near the center of Daihai watershed, which is bordered by Manhan Mountains on the northwest, Matou Mountains on the southeast (Fig 1). The catchment watershed of Daihai Lake is 2312.63 km^2^ and the highest peak in the catchment is Manhan Mountains with elevation of 2106 m. The elevation of Matou Mountains is less than 2042m. The southwest catchment is relatively flat with an elevation of less than 1592 m. According to the field measured data in April 2018, the surface area of Daihai Lake is 51.98 km^2^ with the coastline is about 39.68 km long. The maximum depth of Daihai Lake is 6 m and the water storage capacity is 195 million m^3^.

Daihai Lake is under influence of the mid-temperate semi-arid climate, and the annual average temperature is 5.6°C with average temperature 20°C in summer and -10°C in winter, respectively. The freezing period is from mid-October to mid-April of the following year. The annual sunshine time is 3000 h ~ 3200 h, and the average wind speed is about 4 m/s. Seasons were defined as follows: spring (March to May), summer (June to August), autumn (September to November), and winter (December to February). In winter and spring, the wind direction is mainly northwest and north, and in other seasons, south and southeast wind are dominant. The annual average rainfall is 409.71 mm, and precipitation events mainly concentrate in June to August which is accounting for about 65% of the annual rainfall. The annual average potential evaporation is 1486.42 mm, and the evaporation below 0°C accounts for 18% of the annual evaporation. A total of 22 rivers can recharge Daihai Lake. According to the historical data, the larger perennial rivers are Gongba River, Wuhao River, Buliang River, Tiancheng River and Muhua River, while in recent years, only Buliang River has water all year round. A total of 11 reservoirs have been constructed during 1962~2000, while only 1 reservoir currently hold water, and the other reservoirs do not operate.

Geologically, the Daihai watershed is a narrow and long subsided basin with NEE-SWW trending. The long axis and short axis of the Daihai watershed are about 45 km and 14 km respectively. Daihai watershed was formed during the crustal movement from Pliocene to Quaternary and it began to form a lake in the Early Pleistocene. The main fault zones in this area are NE and NNE trending faults formed during the Yanshan movement, which directly control the geomorphology of this area.

### Data sources

The daily monitoring data of potential evaporation, rainfall and lake water level from 1989 to 2018 were collected from Sansumu hydrological station. The potential evaporation was measured using a 20 cm-diameter evaporation pan, and evaporation of Daihai Lake surface was converted from potential evaporation with conversion coefficient of 0.6 according to local climate characteristics [14]. The daily data of 8 long-term observation wells from December 2015 to December 2018 and the data water consumption of DHPP were collected from Ulanchabu Hydrological Bureau, Inner Mongolia Autonomous Region. The distance between long-term observation wells and Daihai Lake were ranging from 1.31 km to 22.55 km. The depth-area-volume data of Daihai Lake was measured by Yellow River Engineering Consulting Co., Ltd. (YREC) in April 2018.

STRM DEM data with a spatial resolution of 30 m downloaded from the USGS website (https://earthexplorer.usgs.gov/) was used in the present study. Landsat-5 TM satellite and Landsat-8 OLI_TRIS satellite were obtained from the United States Geological Survey website (http://glovis.usgs.gov/). The necessary image preprocessing steps included radiation calibration and atmospheric correction were carried out through ENVI 5.3 software. The image data of land use/land cover (LULC) in eight periods of the Daihai watershed area during 1986~2018 were shown in Table 1. The land use type consisted of grassland, woodland, farmland (including irrigation land and dryland), construction land, water body and bareland. To evaluate the accuracy of the land use map and clarify the vagueness in the initial interpretation, more than 500 digital photos were taken for different types of land use and land cover on the 800 km field verification route. The qualitative accuracy of the completed land use map was more than 95%. For the spatial variation of Daihai Lake, the present study selected 7 images about four to five years apart from 1989 to 2018 and the production time of images were chosen as far as possible from October because of the water storage was relatively stable during this period. Based on Landsat TM/OLI remote sensing images in the period of 1989~2018, the vectorized boundary of Daihai Lake was obtained by artificial visual interpretation technology and the geometric center (also called centroid) of Daihai Lake was calculated using ArcGIS 10.2.

**Table 1:** Remote sensing data used in LULC and Daihai Lake.

Data type	Date	Path/Raw	Resolution	Data source
LULC	1988/9/26, 1993/7/13, 1998/8/28, 2003/7/9,2008/7/6	126/32	30m	Landsat 5(TM)
	2013/9/6, 2018/9/20	126/32	30m	Landsat 8(OLI)
Daihai Lake	1989/10/6,1993/10/17,1998/10/15,2003/10/29, 2008/10/10	126/32	30m	Landsat 5(TM)
	2013/10/8, 2018/10/6	126/32	30m	Landsat 8(OLI)

### Statistical method

#### Mann-Kendall trend test

The Mann-Kendall trend (MKT) test method is a non-parametric test method recommended by the World Meteorological Organization (WMO) and has been widely used in meteorological and hydrological time series data to detect significant trend [17]. The MKT test does not require the assumption of normality or homogeneity of variance [18]. This test compares median rather than mean and, as a result, if the data have one or two outliers, their influence is neglected. The MKT test statistic (S) is defined as [18]:

$$S=\sum_{i=1}^{n-1}\sum_{j=i+1}^{n}\text{sgn}\left(X_{j}-X_{i}\right)$$ (1)

where n is the number of observations, X_i_ is the rank for ith observations (i = 1, 2…n − 1), X_j_ is the rank for jth observations (j = i + 1, 2…n), and sign function is computed as:

$$\text{sgn}\left(X_{j}-X_{i}\right)=\left\{\begin{array}{cc} 1 & \left(X_{j}-X_{i}\right)>0\\ 0 & \left(X_{j}-X_{i}\right)=0\\ -1 & \left(X_{j}-X_{i}\right)<0 \end{array}\right.$$ (2)

The test statistic S is assumed asymptotically normally distributed for the series where sample size n ≥10 with mean E(S) and variance Var (S) as:

E(S)=0 (3)

$$Var(S)=\frac{n(n-1)(2n+5)-\sum_{i=1}^{P}t_{i}\left(t_{i}-1\right)\left(2t_{i}+5\right)}{18}$$ (4)

where P is the number of tied groups (a tied group is a sample data having the same value), and t_i_ is the number of data values in the ith tied group (i = 1,2,3…P).

After the calculation of Var(S) of time series data, the standardized Z_MKT_ value is calculated using the following equation:

$$Z_{MKT}=\left\{\begin{array}{cc} \frac{S-1}{\sqrt{Var(S)}} & \text{S}>0\\ 0 & S=0\\ \frac{S+1}{\sqrt{Var(S)}} & S<0 \end{array}\right.$$ (5)

The Z_MKT_ follows the standard normal distribution with mean zero (μ = 0) and variance one (σ^2^ = 1). A positive (negative) value of Z_MKT_ indicates an increasing (decreasing) trend in the time series. Given a confidence level α, the sequential data would be supposed to experience statistically significant trend if |Z_MKT_|>Z_(1-α/2),_ where Z_(1-α/2)_ is the corresponding value of α/2 following the standard normal distribution. The hypothesis of an upward or downward trend cannot be rejected at the α = 0.05 if | Z_MKT_|≥1.96.

Besides, the magnitude of a time series trend was evaluated by a simple non-parametric procedure developed by Sen [19]. The trend is calculated by

$$\beta =median\left(\frac{X_{j}-X_{i}}{j-i}\right),\;j>i$$ (6)

where *β* is Sen’s slope estimate. *β* > 0 indicates a upward trend in a time series. Otherwise, the data series presents a downward trend during the period.

#### Mann–Kendall–Sneyers test

Mann-Kendall- Sneyers (MKS) test can be used to detect the abrupt changes of climate and hydrological data. Abrupt climate change is defined as a phenomenon in which the climate jumps from a stable state (or a stable and sustained change trend) to another stable state (or a stable and sustained change trend) [20]. Given that x_1_, x_2_…, x_n_ is a series that denotes the time series studied, and n is the length of the sequence, the order column S_k_ is constructed as follows [21]:

$$S_{k}=\sum_{i=1}^{k}\sum_{j}^{i-1}a_{ij}\;(k=2,3,\cdots ,n)$$ (7)

$$a_{ij}=\left\{\begin{array}{ll} 1 & x_{i}>x_{j}\\ 0 & x_{i}\leq x_{j} \end{array},(1\leq j\leq i)\right.$$ (8)

Statistical variables are defined as

$$\text{UF}(\text{k})=\frac{S_{k}-E\left(S_{k}\right)}{\sqrt{Var\left(S_{k}\right)}}$$ (9)

in which

$$E\left(S_{k}\right)=\frac{k(k+1)}{4}$$ (10)

$$Var\left(S_{k}\right)=\frac{k(k-1)(2k+5)}{72}$$ (11)

The data sequence x is arranged in reverse order. Then, according to Eq (9),

$$\left\{\begin{array}{c} UB(k)=-UF\left(k^{\prime }\right)\\ {\text{k}}^{\prime }=\text{n}+1-\text{k} \end{array},(k=1,2,\cdots ,n)\right.$$ (12)

where UF(k) and UB(k) are the forward and backward sequences, respectively.

UF(k) and UB(k) are plotted to locate the beginning of the change at the intersection between the curves [22]. If the intersection occurs within the confidence interval, it indicates a mutation point. The significance level in this study was 0.05, and the critical values were U_0.05_ = ± 1.96. If the intersection is outside the confidence interval, another method (a moving t-test technique was used for this study) is needed to analyze the data again.

#### Moving t-test

The moving t-test technique is adopted to detect mutation point by evaluating significant difference between two samples, and has been extensively used. This method divides one random variable into two consecutive subsets *x*_1_ and *x*_2_, where *u*_i_, *s*_i_^2^ and *n*_i_ represent the mean value, variance and sample size of *x*_i_ (i = 1, 2), respectively. The procedures are as follows [21]:

Null hypothesis: *H*_0_: *u*_1_ – *u*_2_ = 0. Statistic *t*_0_ is defined by

$$t_{0}=\frac{x_{1}-x_{2}}{S_{p}\sqrt{\frac{1}{n_{1}}+\frac{1}{n_{2}}}}$$ (13)

where S_p_ is:

$$S_{p}=\sqrt{\frac{n_{1}s_{1}^{2}+n_{2}s_{2}^{2}}{n_{1}+n_{2}-2}}$$ (14)

*t*_0_ complies with the t-distribution with degree of freedom *v* = *n*_1_ + *n*_2_−2. The null hypothesis would be rejected once |*t*_0_ | ≥ *t*_α_ at a significant level α (α = 0.05 in the present study). It means that there is an abrupt change in the sample series. Because different choice of the subsets length can affect the location of mutation point, two conditions of *n*_1_ = *n*_2_ = 7 and *n*_1_ = *n*_2_ = 10 were chosen in this study.

#### Multi linear regression

Multiple linear regression (MLR) attempts to model the relationship between two or more explanatory variables and a response variable by fitting a linear equation to observed data [23]. It helps in determining the level of variation between the variables. MLR line of Y (dependent variable) on X (independent variable) defined in the Eq (15).

$$\text{y}=b_{0}+b_{1}x_{1}+b_{2}x_{2}+\cdots +b_{i}x_{i}$$ (15)

Where *x*_1_ is the value of the *i*th predictor, *b*_*0*_ is the regression constant, and *b*_*i*_ is the coefficient of the *i*th predictor.

## Results

### Variation of Daihai Lake

**Fig 2:**
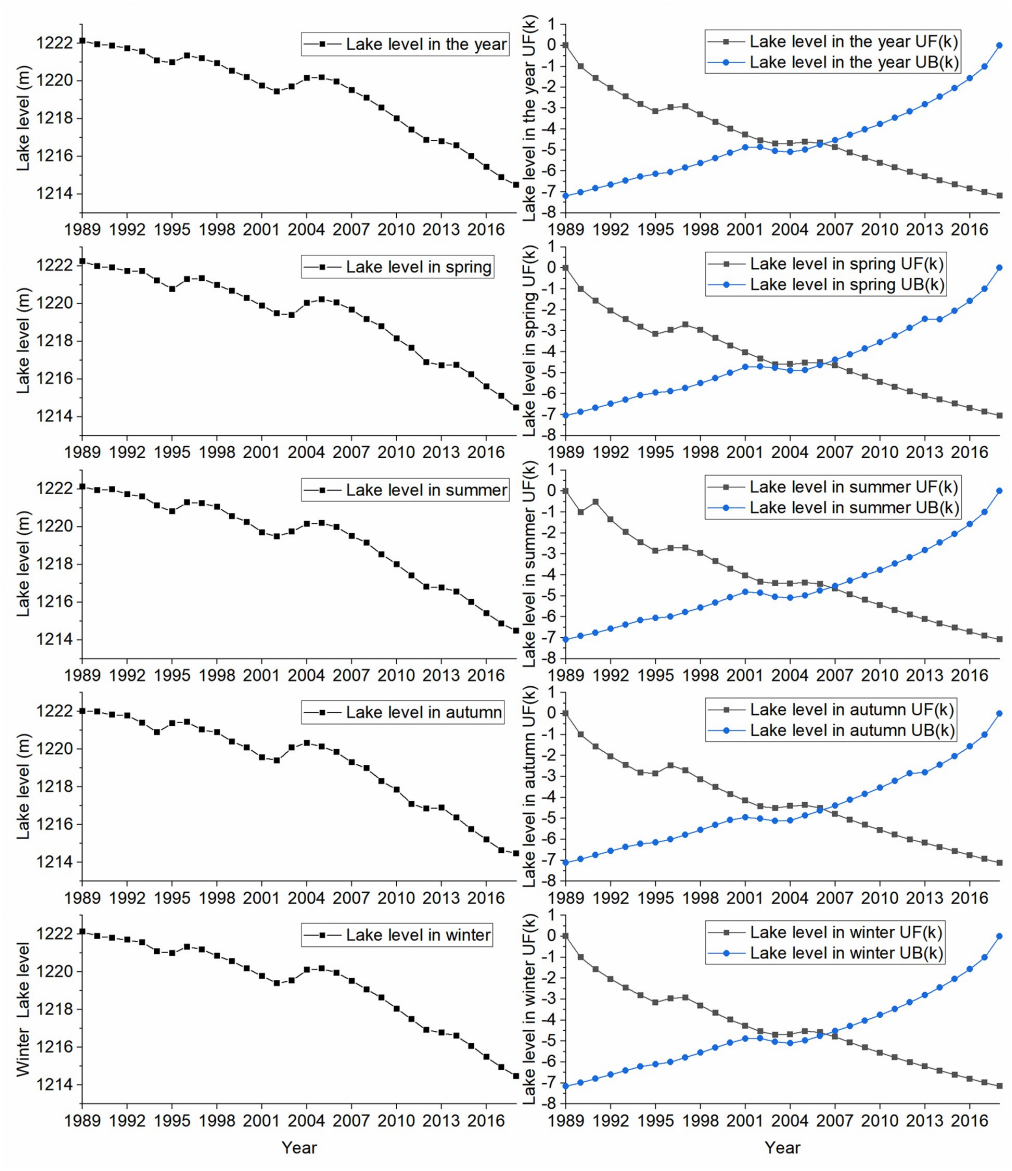
Plot showing variation of Daihai Lake level and the MKS test statistics UF(k) and UB(k).

The lake water level of Daihai Lake showed a significant downward trend (Fig 2), and the annual average water level in 2018 was 7.81 m lower than that in 1989 with a decreasing rate -0.24m/a (Table 2). The annual average lake water level of Daihai Lake was 1219.279 m. In 2011, the lake water level dropped the most with a decline of -0.74 m, following by 2009, 2015 and 2017 with a decline of -0.65 m, -0.61 m and -0.61 m respectively. Three obvious rising processes were observed in 1992, 1995 ~ 1996 and 2003 ~ 2004, and the lake water level rose 0.04m, 0.54m and 0.95m respectively.

**Table 2:** Results of the MKT test and moving t-test of lake level.

Time Scale	Specific scale	Z_MKT_	Trend	Sen’s slop	n_1_ = n_2_ = 7		n_1_ = n_2_ = 10	
					t_0_	t_0.05_	t_0_	t_0.05_
Annual	1989~2018	-7.17*	Downtrend	-0.24	/	/	/	/
	Pre-2006	-4.62*	Downtrend	-0.14	3.11*	2.56	4.92*	2.45
	Post-2006	-4.46*	Downtrend	-0.45				
Spring	1989~2018	-7.03*	Downtrend	-0.24	/	/	/	/
	Pre-2006	-4.47*	Downtrend	-0.15	2.76*	2.56	4.61*	2.45
	Post-2006	-4.32*	Downtrend	-0.46				
Summer	1989~2018	-7.07*	Downtrend	-0.24	/	/	/	/
	Pre-2006	-4.39*	Downtrend	-0.14	3.09*	2.56	4.91*	2.45
	Post-2006	-4.46*	Downtrend	-0.46				
Autumn	1989~2018	-7.1*	Downtrend	-0.25	/	/	/	/
	Pre-2006	-4.47*	Downtrend	-0.14	3.46*	2.56	5.24*	2.45
	Post-2006	-4.32*	Downtrend	-0.45				
Winter	1989~2018	-7.14*	Downtrend	-0.24	/	/	/	/
	Pre-2006	-4.55*	Downtrend	-0.14	3.03*	2.56	4.83*	2.45
	Post-2006	-4.46*	Downtrend	-0.46				

Note: Z_MKT_: statistic of MKT test;

*t*_0_: statistic of moving t-test;

*t*_0.05_: critical value of t-distribution at a significant level 0.05;

n_1_, n_2_: sample size of moving t-test;

*: results showed significant trend at significant level 0.05.

The decreasing order of seasonal average lake water level was as follows: spring (1219.346 m) > summer (1219.284 m) > winter (1219.277 m) > autumn (1219.209 m). Results of Kruskal Wallis nonparametric test [24] showed that only spring and autumn had significant difference (p-value = 0.019). The average lake water level in spring and summer were both higher than the annual average lake water level, while average lake water level in autumn and winter were lower than the annual average lake water level.

The results of MKS test and moving-t test (Table 2) showed that the annual average lake water levels in 2006 was an obvious mutation point at all time scales (annual, spring, summer, autumn and winter), the Sen’s slope of lake water in pre-2006 and post-2006 were -0.14 m/a and -0.46 m/a, respectively. The descending speed of Daihai Lake water level in post-2006 was 3.22 times that of pre-2006.

**Fig 3:**
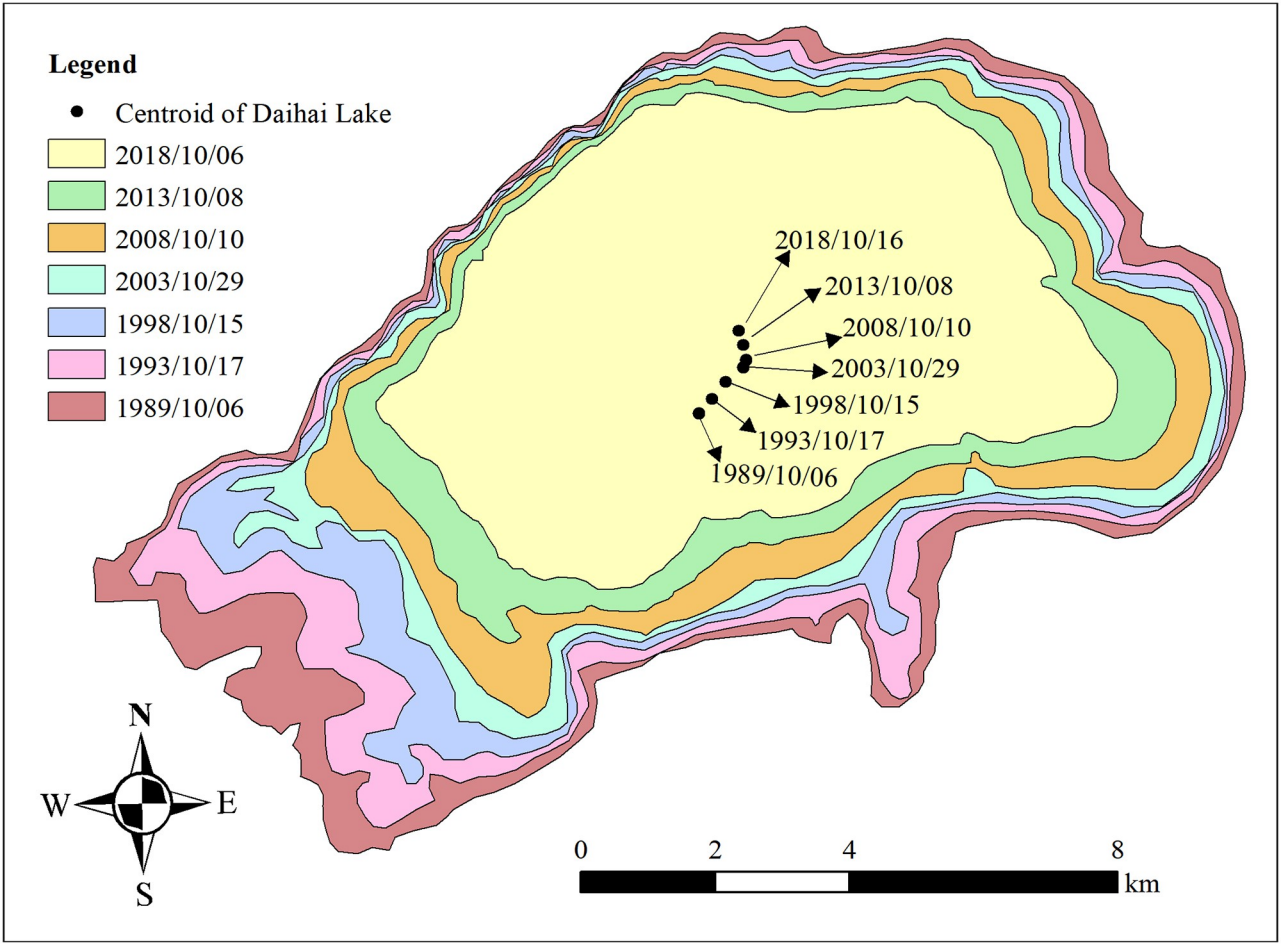
Spatial variation of Daihai Lake surface.

According to the results of remote sensing data, the area of Daihai Lake in 1989, 1993, 1998, 2003, 2008, 2013 and 2018 were 115.09 km^2^, 103.37 km^2^, 92.6 km^2^, 83.73 km^2^, 76.27 km^2^, 64.23 km^2^ and 51.92 km^2^ respectively. The shrinkage rate of Daihai Lake area decreased from -2.34 km^2^/a during 1989~1993 to -1.49 km^2^/a during 2003~2008 and increased to -2.46 km^2^/a during 2013~2018 (Table 3). The shrinkage rate in the last 5 years of Daihai Lake area was the highest in last 30 years, following by the period 2008~2013. The centroid of Daihai Lake moved 1365.18 m from southwest to northeast during 1989 ~ 2018 with an average speed of 47.08 m/a (Fig 3). Specifically, the centroid of Daihai Lake first moved from southwest to northeast during 1989~2003 and then moved to northwest during 2003~2018. In the past three decades, the moving speed of centroid peaked at 72.14 m/a during 1989~1993.

**Table 3:** Statistical results of variations of Daihai Lake.

Periods	Area variation (m^2^)	Rate of area variation (m^2^/a)	Moving distance of centroid (m)	Moving speed of centroid (m/a)
1989~1993	-11.72	-2.34	288.58	72.14
1993~1998	-10.78	-2.16	323.17	64.63
1998~2003	-8.87	-1.77	344.87	68.97
2003~2008	-7.46	-1.49	119.26	23.85
2008~2013	-12.04	-2.41	224.96	44.99
2013~2018	-12.31	-2.46	219.44	43.89

### Characteristics of climate variability

#### Variation of lake evaporation

**Fig 4:**
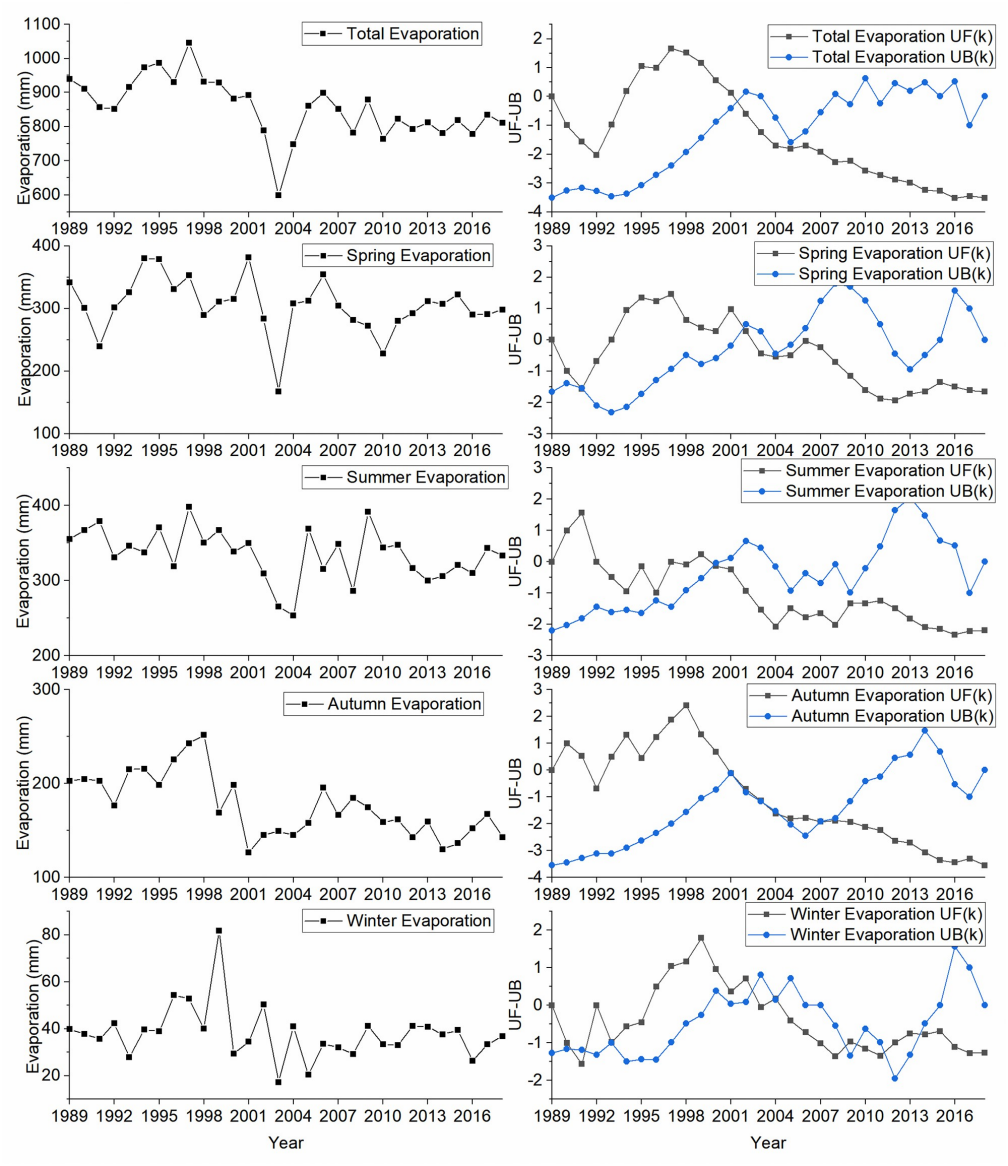
Plot showing evaporation variation and the MKS test statistics UF(k) and UB(k).

The annual average evaporation was 855.58 mm in the last three decades. The MKT test revealed that the annual evaporation showed a significant downward trend (Z_MKT_ = - 3.5) with a rate of approximately -5.33 mm/a (Table 4). A mutation point was found in 2002 (Fig 4), but no significant trend pre-2002 and post-2002 were observed (|Z_MKT_| < 1.96) respectively.

**Table 4:** Statistical results of evaporation variation in Daihai Lake.

Time scale	Mutation points	Specific scale	Z_MKT_	Trend	Sen’s slop
Annual	2002	1989~2018	-3.5*	Downtrend	-5.33
		Pre-2002	0.06	No	1.26
		Post-2002	0.12	No	1.73
Spring	2002	1989~2018	-1.64	No	-1.31
		Pre-2002	0.22	No	0.54
		Post-2002	0.23	No	0.89
Summer	2000	1989~2018	-2.18*	Downtrend	-1.43
		Pre-2000	-0.07	No	-0.71
		Post-2000	0.08	No	0.01
Autumn	2001,2007	1989~2018	-3.53*	Downtrend	-2.22
		Pre-2001	0.62	No	2.75
		2001~2007	2.1*	Downtrend	6.08
		Post-2007	-1.71	No	-3.34
Winter	2003,2014	1989~2018	-1.25	No	-0.15
		Pre-2003	0.66	No	0.51
		2003~2014	1.40	No	1.82
		Post-2014	-0.24	No	-0.53

Note: Z_MKT_: statistic of MKT test.

The decreasing order of seasonal average evaporation was summer (335.65mm) > spring (305.18mm) > autumn (176.71mm) > winter (38.05mm), and the evaporation in summer was 8.82 times higher than that of winter. A significant downward trend was found in both summer and autumn (|Z_MKT_ | > 1.96) with downward rates -1.43 mm/a and -2.22 mm/a, respectively. Evaporation in different seasons showed significant differences using Kruskal Wallis nonparametric test (p-value < 0.001). The mutation points of evaporation occurred in 2000, 2001, 2002, 2003, 2007 and 2014 in seasonal serial, but only the evaporation in autumn increased significantly from 2001 to 2007 (Z_MKT_ = 2.1).

The annual, summer, autumn and winter evaporation in arid and semi-arid areas of North China shared the same trend as observed in Daihai Lake [25, 26]. Changes in climate, including air temperature, humidity, wind speed, and available energy, can have an important influence on long-term trends in lake evaporation [27]. As the annual temperature of Daihai watershed showed a significant increasing trend, rising, the decreasing trend of Daihai Lake evaporation may be due to wind speed decreased, similar to arid region lakes [8].

#### Variation of lake precipitation

**Fig 5:**
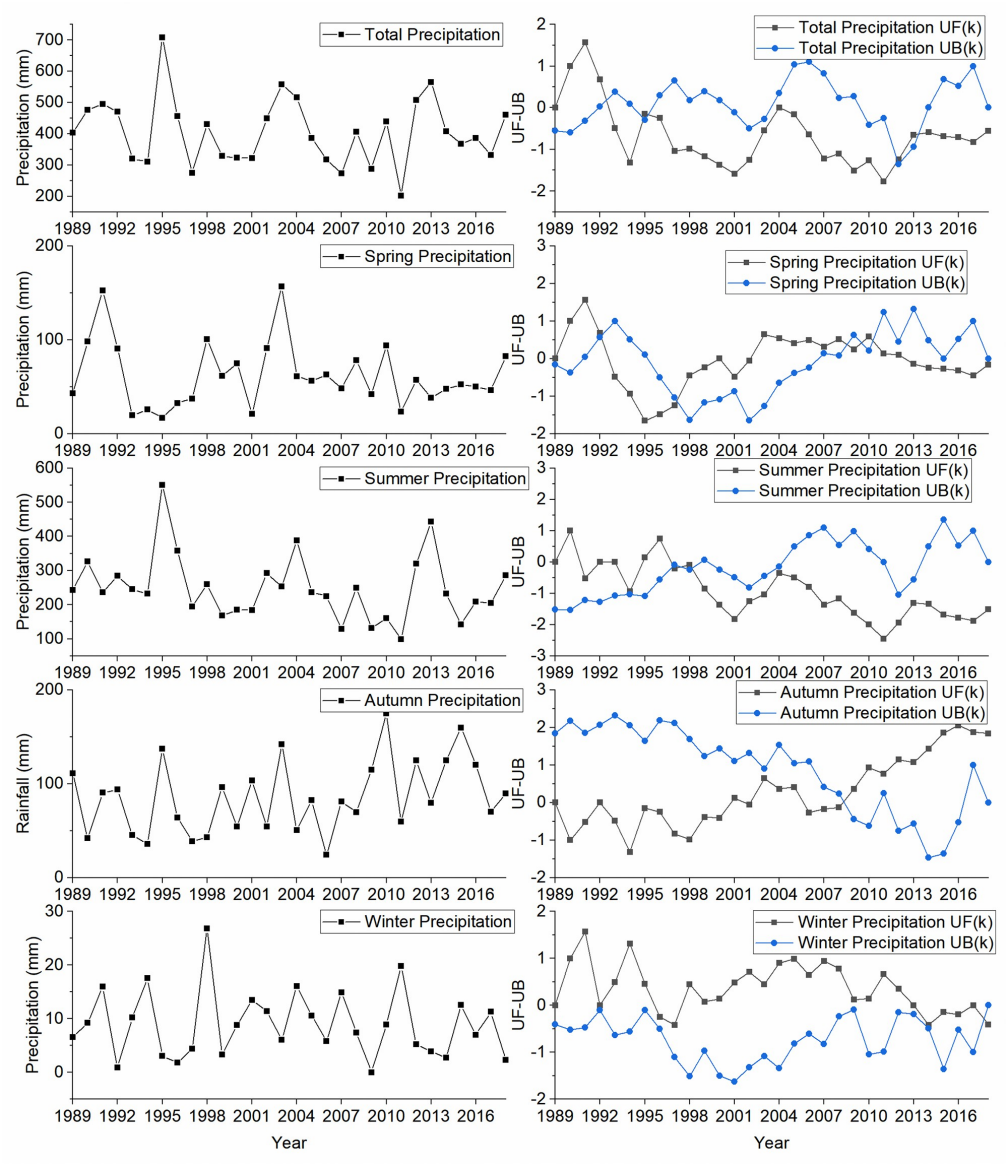
Plot showing precipitation variation and the MKS test statistics UF(k) and UB(k).

The annual average precipitation of Daihai Lake was 405.96 mm in last 30 years, while no significant trend (Z_MKT_ = - 0.54) was found using the MKT test (Table 5). The decreasing order of seasonal average precipitation was summer (248.96 mm) > autumn (85.98 mm) > spring (62.08 mm) > winter (8.94 mm), and the precipitation in summer was 27.85 times higher than that of winter. Precipitation in different seasons showed significant differences using Kruskal Wallis nonparametric test (p-value < 0.001). The mutation points of precipitation in different time series were observed in 1995, 1998, 2009, 2010 and 2013 (Fig 5), but no significant trend was observed before and after all these mutation points (|Z_MKT_| < 1.96). Except for winter, the characteristics of precipitation in the arid and semi-arid areas of North China in all time scales were similar to those of Daihai Lake [25, 26].

**Table 5:** Statistical results of precipitation variation in Daihai Lake.

Time scale	Mutation points	Specific scale	Z_MKT_	Trend	Sen’s slop
Annual	1995,2013	1989~2018	-0.54	No	-1.43
		Pre-1995	-1.13	No	-21.03
		1995~2013	-1.14	No	-5.64
		Post-2013	-0.75	No	-20.94
Spring	1998,2010	1989~2018	-0.14	No	-0.17
		Pre-1998	-0.36	No	-3.42
		1998~2010	-0.62	No	-2.02
		Post-2010	0.10	No	1.63
Summer	1998	1989~2018	-1.50	No	-2.82
		Pre-1998	-0.10	No	-1.85
		Post-1998	-0.21	No	-0.83
Autumn	2009	1989~2018	1.82	No	1.49
		Pre-2009	-0.10	No	-0.42
		Post-2009	-0.36	No	-2.82
Winter	No	1989~2018	-0.39	No	-0.05

Note: Z_MKT_: statistic of MKT test.

### Land use change

**Fig 6:**
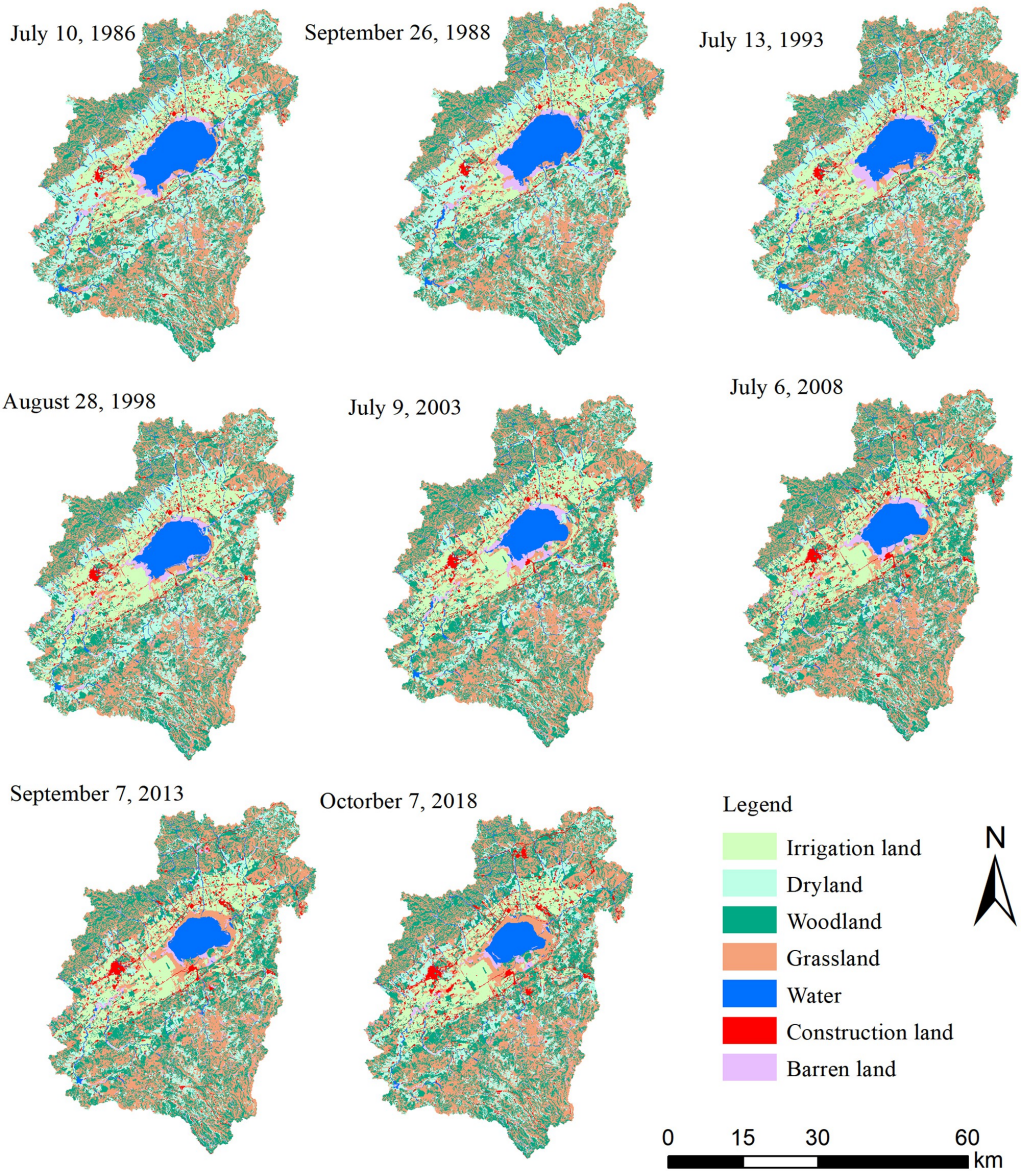
Spatiotemporal pattern of land use types in Daihai wetland during 1986~ 2018.

Grassland, woodland and farmland (including irrigation land and dryland) were the main types of land use in Daihai watershed (Fig 6). Grasslands and woodland were mainly distributed in mountainous and hilly areas, such as Manhan and Matou Mountains. Farmland and construction land were mainly distributed in low-lying and flat areas in the middle of the Daihai wetland. Water bodies mainly distributed in the middle of the Daihai wetland and consisted of Daihai Lake, rivers, reservoirs and beach land. Barren land was scattered, mainly in areas such as the edge of Daihai Lake. Grassland, woodland and farmland together accounted for 87.36%~89.57% (Table 6) of Daihai wetland during 1986~2018, and the area of water bodies, construction land, and bareland accounted for 10.43–12.64% of the total Daihai wetland area.

**Table 6:** LULC in Daihai wetland during 1986–2018 (Unit: km^2^).

Year	Statistical item	Woodland	Grassland	Irrigation land	Dryland	Water body	Construction land	Barren land
1986	Area	531.90	725.06	205.79	557.46	174.15	57.75	60.52
	%	23.00%	31.35%	8.90%	24.11%	7.53%	2.50%	2.62%
1988	Area	533.64	724.13	208.18	556.63	168.03	58.92	63.10
	%	23.08%	31.31%	9.00%	24.07%	7.27%	2.55%	2.73%
1993	Area	548.56	739.84	293.06	457.22	150.99	60.86	62.08
	%	23.72%	31.99%	12.67%	19.77%	6.53%	2.63%	2.68%
1998	Area	559.35	749.58	331.39	411.87	135.64	63.42	61.38
	%	24.19%	32.41%	14.33%	17.81%	5.87%	2.74%	2.65%
2003	Area	618.05	791.67	342.39	304.70	120.40	69.44	65.98
	%	26.72%	34.23%	14.81%	13.18%	5.21%	3.00%	2.85%
2008	Area	646.94	817.29	349.86	238.31	114.21	82.75	63.28
	%	27.97%	35.34%	15.13%	10.30%	4.94%	3.58%	2.74%
2013	Area	662.00	847.71	302.24	258.26	105.52	86.18	50.73
	%	28.63%	36.66%	13.07%	11.17%	4.56%	3.73%	2.19%
2018	Area	669.22	849.23	246.63	306.30	95.92	99.32	46.01
	%	28.94%	36.72%	10.66%	13.24%	4.15%	4.29%	1.99%

Woodland, grassland and construction land increased gradually from 531.9km^2^, 725.06km^2^ and 57.75km^2^ in 1986 to 669.22 km^2^, 849.23 km^2^ and 99.32 km^2^ in 2018 with increasing rate 4.29km^2^/a, 3.88km^2^/a and 1.30km^2^/a, respectively. Water body decreased gradually from 174.15 km^2^ in 1986 to 95.92 km^2^ in 2018 with decreasing rate -2.44 km^2^/a. Farmland decreased gradually from 763.25 km^2^ in 1986 to 552.93 km^2^ in 2018 with decreasing rate -6.57 km^2^/a. Specifically, irrigation land increased gradually from 205.79 km^2^ in 1986 to 349.86 km^2^ in 2008, then decreased gradually to 246.63 km^2^ in 2018, while dryland decreased gradually from 557.46 km^2^ in 1986 to 238.31 km^2^ in 2008, then increased gradually to 306.30 km^2^ in 2018. The barren land area decreased from 60.52 km^2^ in 1986 to 46.01 km^2^ in 2018. It changed slightly during 1986~2008 ranging from 60.52km^2^~65.98km^2^, while a relative sharp decline of 12.56 km^2^ was observed in 2013 compared to 2008.

The result of LULC transfer matrix (Table 7) showed that a total of 41.01km^2^, 16.62 km^2^, 2.65 km^2^, 11.55km^2^, 6.54 km^2^ and 2.25 km^2^ water body in 1986 transformed into grassland, irrigation land, dryland, barren land, woodland and construction land in 2018, respectively.

**Table 7:** LULC transfer matrix from 1986 to 2018 in Daihai wetland (Unit: km^2^).

LULC	Woodland	Water body	Grassland	Irrigation land	Dryland	Construction land	Barren land	2018
Woodland	520.85	6.54	14.05	9.10	115.37		3.32	669.22
Water body	0.01	93.53	1.32	0.03	0.28		0.75	95.92
Grassland	3.88	41.01	680.74	5.95	105.80		11.85	849.23
Irrigation land	1.35	16.62	6.36	154.98	56.63		10.69	246.63
Dryland	3.21	2.65	10.21	25.67	263.90		0.66	306.30
Construction land	2.27	2.25	11.01	9.95	14.25	57.75	1.84	99.32
Barren land	0.32	11.55	1.39	0.11	1.23		31.40	46.01
1986	531.90	174.15	725.06	205.79	557.46	57.75	60.52	2312.63

### Variation of groundwater level

**Fig 7:**
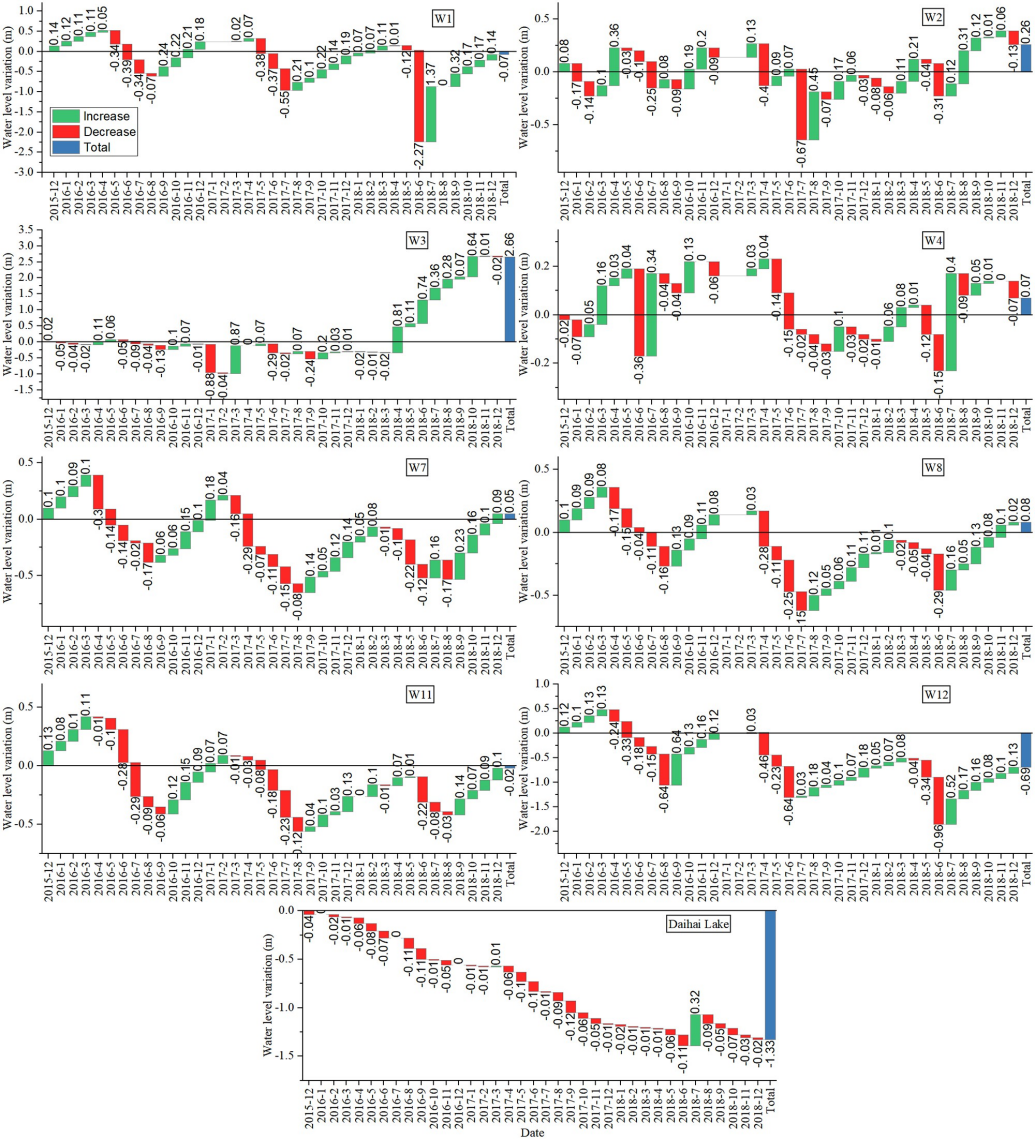
Monthly variation of Daihai Lake level and long-term observation wells.

The monthly variation of Daihai Lake level and long-term observation wells were calculated as shown in Fig 7. W1, W7, W8, W11 and W12 shared similar variation trends that the groundwater level in these wells showed a downward trend from May to August and an upward trend in other months. There was no obvious trend in W2, W3 and W4. Compared with December 2015, groundwater levels of W2, W3, W4, W7 and W8 increased by 0.26 m, 2.66 m, 0.07 m, 0.05 m and 0.08 m respectively in December 2018, while W1, W11 and W12 decreased by 0.07 m, 0.02 m and 0.69 m respectively.

Daihai Lake level in March 2017 and July 2018 increased by 0.01m and 0.32m, respectively, and the remaining months showed a downward trend. In March 2017, W1, W2, W3, W4, W8 and W12 increased by 0.02 m, 0.13 m, 0.87 m, 0.03 m and 0.03 m respectively, while W7 and W11 decreased by 0.16 m and 0.01 m respectively. In July 2018, W1, W2, W3, W4, W7, W8 and W12 increased by 1.37 m, 0.12 m, 0.36 m, 0.4 m, 0.16 m, 0.16 m and 0.52 m respectively, while W11 decreased by 0.08 m.

## Discussion

### Impact of climate variability

Precipitation (including solid precipitation) and evaporation are most important factors influencing the water budget of closed lakes. The correlation analysis through Spearman method showed that evaporation had a very weak relationship with lake water level (correlation coefficient r = 0.078, p < 0.01) and precipitation (r = -0.038, p < 0.01), respectively, and the precipitation showed no significant correlation with the lake water level. In order to reduce the interference of irrigation activities (from May to August) on lake water level, the data from May to August were excluded and the correlation analysis was conducted again. The results showed that the correlation coefficients of evaporation with lake water level and precipitation were 0.108 and -0.075 (p-value < 0.01), respectively, and the precipitation showed no significant correlation with lake water level. It can be seen that without the irrigation period data, the correlation between evaporation and lake water level, precipitation increased slightly but still very weak, and there was still no significant correlation between precipitation and lake water level. These results suggested that the reasons for shrinkage of Daihai Lake were complex and the non-climate factor were more and more important.

### Impact of human activities

The population, gross domestic product (GDP) and food production of Liangcheng County increased from 23.8×10^4^, 58393×10^4^ yuan and 159124 ton in 1999 to 24×10^4^, 781030×10^4^ yuan and 251430 ton in 2016 respectively [28, 29]. The population of Liangcheng increased slightly, while the GDP and food production in 2016 were 13.38-fold and 1.58-fold than that of 1999, respectively. The increase in population, GDP and food production lead to a higher water demand and exacerbated the contradiction between water demand and water resources. Daihai Lake was affected by human activities mainly from LULC, building reservoirs, pumping groundwater and directly consuming Daihai Lake water by Daihai power plant. Different human activity in Daihai wetland affects local hydrological circle in different ways, while they all play an important role in the variation of Daihai lake level.

LULC is one of the most important factors affecting Daihai Lake, and it is the comprehensive embodiment of the influence of human activities on Daihai Lake. Land use analysis showed that woodland and grassland in the study area increased significantly, while farmland was opposite. The reasons of these findings were as follows: Firstly, the Three-North Shelterbelt Project launched in late 1970s and ecological protection activities, such as grassland protection and afforestation in Liangcheng County, were strengthened; Secondly, a large amount of farmland reclaimed by grassland and woodland because the national project of returning farmland to grassland and forests since 2000; Last but not least, The local government closed about 140 km^2^ of irrigation land since 2017 as the Daihai Lake shrank continually. Water body showed significant negative relationship with woodland, grassland and construction land, and positive relationship with dryland (Table 8). As the dryland is not irrigated by local farmer, it may be positive to the recovery of Daihai Lake. Woodland and grassland are mainly distributed in the surrounding mountainous areas which are the main catchment area of the Daihai wetland, the increase of woodland and grassland may not conducive to the formation of surface runoff and increased evapotranspiration. The increase of construction land played a considerable role in hydrological dynamics with changes on actual transpiration, base flow, runoff, percolation and soil water [30].

**Table 8:** The relationship between different land use types.

**LULC**	**Woodland**	**Grassland**	**Irrigation land**	**Dryland**	**Water body**	**Construction land**	**Barren land**
**Woodland**	1						
**Grassland**	0.994**	1					
**Irrigation land**	0.445	0.395	1				
**Dryland**	-0.934**	-0.910**	-0.733*	1			
**Water body**	-0.962**	-0.959**	-0.565	0.945**	1		
**Construction land**	0.948**	0.960**	0.223	-0.804*	-0.914**	1	
**Barren land**	-0.596	-0.671	0.259	0.341	0.572	-0.771*	1

**. Correlation is significant at the 0.01 level (2-tailed).

*. Correlation is significant at the 0.05 level (2-tailed).

The construction of reservoirs is another representative anthropogenic activity in the Daihai wetland. The local government began to build reservoirs, such as the Shuanggucheng, Gongba, Bianfeng, Shimen, Wuhao, and Shiyuegou reservoirs, for power generation and irrigation in 1960s and large-scale farmland infrastructure were developed in the following decades. The agricultural water consumption increased rapidly from of 3457×104 m^3^/a in the 1960s to 4824×10^4^ m^3^/a in the 1970s [31], and peaked at approximately 6251×104 m^3^/a during the 1980s. In 2000, a total of 11 reservoirs have been constructed with total water storage to approximately 3832×10^4^ m^3^ [14]. These reservoirs reduced the surface runoff of Daihai Lake and increased open water evaporation from their surfaces. At present, only the Wuhao River reservoir still has a small amount of storage, while all the others have run out of water and have been transformed into either grasslands or farmland due to decreased runoff. However, it is worth noting that the reservoirs built in Daihai wetland were operated from August to march of the next year. Due to the small storage capacity, most of the peak flow flowed directly into the lake during the flood period. In dry years, the proportion of detained water in the total runoff increases significantly, which aggravated the water shortage of Daihai Lake.

Groundwater is another important water source of Daihai Lake and local farmer pumped groundwater for irrigation from May to August. The groundwater level showed an obvious downward trend during the irrigation period, indicating that the groundwater level around Daihai was affected by irrigation activities noticeably. The rose of Daihai Lake level in March 2017 and July 2018 were highly consistent with the rose of the surrounding groundwater level. This result indicates the close relationship between groundwater and lake water, while the lake water–groundwater interaction in the Daihai watershed is complicated and weakly studied in the past. Therefore, more detailed evidence was needed to shed light on the lake water–groundwater relationship.

DHPP was located to the south of Daihai Lake and it began to use Daihai Lake water for cooling the facility since 2006. In early years, the DHPP directly consumed 800×10^6^ m^3^ (including additional evaporation loss by thermal water) of lake water while the amount increased to 1.192×10^7^ m^3^ since 2010. However, with the rapid decline of Daihai Lake water level in recent years, DHPP reduced the water consumption of Daihai Lake to 800× 10^6^ m^3^ in 2018 through energy-saving and emission reduction measures and not used Daihai Lake water after 2019 years. It was thought-provoking that DHPP began to consume Daihai lake water in 2006, which was consistent with abrupt change of Daihai lake level. The consumption of Daihai Lake by DHPP had great impact on Daihai Lake and this was direct evidence that human activities affected the lake water level.

### Quantitative contribution of climate and human activity

Quantitative evaluation of the effect of climate variability and human activities on runoff is of great importance for water resources planning and management in terms of maintaining the ecosystem integrity and sustaining the society development. Nonetheless, quantifying the individual effects of climate variability and human activities on hydrological regime is a challenge. Since there were no monitoring stations on the rivers around Daihai, multi-year recharge data were not available. According to practical situation of Daihai lake, multi-linear regression analysis method and water balance method were used to separate the impacts of climate variability and human activities.

**Fig 8:**
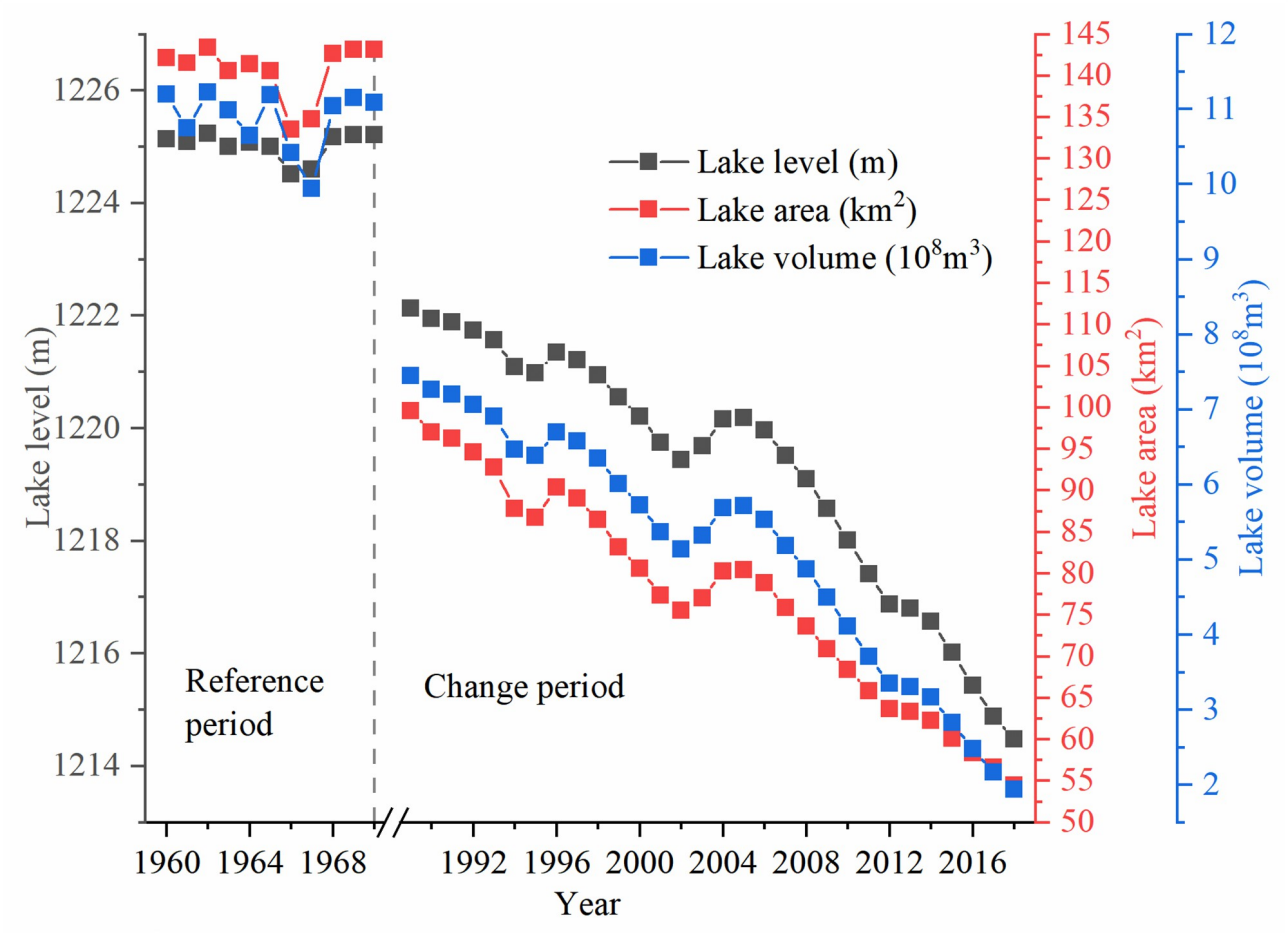
The relationship of Daihai Lake depth-area-volume.

The present study assumed that Daihai Lake was not affected by human activities in the 1960s because the human activities around Daihai Lake were relatively weak at this period [32]. The period of 1960~1970 were selected as reference period and the data of precipitation, evaporation and lake level of Daihai lake in this period were collected from Huang [32]. In reference period, the recharge items of Daihai Lake were precipitation, river water and groundwater, and the discharge item was evaporation. Lake depth-area-volume relationships were established using lake bathymetries (Fig 8). Total amount of lake surface evaporation(TALE), total amount of lake surface precipitation (TALP), volume change of lake (VCL) and total amount of recharge into the lake including surface water and groundwater with no human disturbance (Inflow_No_Human_) were calculated respectively (Table 9) by the following equations:

TALE=Evaporation×Area (16)

TALP=Precipitation×Area (17)

$$VCL=VL_{end\;of\;the\;year\;}-VL_{beginning\;of\;the\;year\;}$$ (18)

$$Inflow_{No-Human\;}=TALE-TALP+VCL$$ (19)

**Table 9:** The statistical information of Daihai Lake in reference period.

Year	ACLL (m)	Precipitation (mm)	Evaporation (mm)	Lake area (km^2^)	TALP (10^4^m^3^)	TALE (10^4^m^3^)	VCL (10^4^m^3^)	*Inflow*_*No_Human*_ (10^4^m^3^)
1960	-0.32	285.7	873.18	142.13	4089.62	12499.04	-4522.56	3827.55
1961	0.34	596	863.58	141.51	8278.25	11994.85	4810.15	8596.74
1962	-0.17	294.8	792.12	143.40	4227.40	11358.92	-2435.63	4695.89
1963	-0.24	323.8	729.42	140.53	4574.19	10304.21	-3337.21	2363.05
1964	0.38	581.8	746.82	141.39	8021.47	10296.65	5341.57	7674.77
1965	-0.55	201	1206.3	140.53	2874.64	17252.10	-7665.40	6462.28
1966	-0.36	375.3	1322.4	133.50	5083.15	17910.90	-4789.11	7854.48
1967	0.81	588.1	1104	134.74	7666.87	14392.49	11021.44	17972.79
1968	0.08	425.5	1201.08	142.64	6031.91	17026.57	1145.90	12208.53
1969	-0.05	479.6	1229.76	143.14	6846.92	17556.45	-716.71	10021.37
1970	0.05	498.4	1126.26	143.14	7083.98	16008.03	716.71	9704.14

Note: ACLL: annual change of lake level

TALP: total amount of lake precipitation;

TALE: total amount of lake evaporation;

VCL: volume change of lake;

*Inflow*_*No_Human*_: total amount of recharge into the lake including surface water and groundwater with no human disturbance.

Where Evaporation is annual lake evaporation (mm), Precipitation is annual lake precipitation (mm), Area is the annual lake area (km^2^), VL_end of the year_ is volume of lake at the end of the year (10^4^m^3^), VL_beginning of the year_ is volume of lake at the beginning of the year(10^4^m^3^).

A linear relationship between the Inflow_No-Human_ (unit: 10^4^m^3^) and annual lake evaporation (unit: mm), annual lake precipitation (unit: mm) was established using the data from 1960 to 1970. The linear fitting equation was shown in Eq (20) and correlation efficient R^2^ was 0.726, which showed the fitting effect was good.


$$Inflow_{No_{-}Human\;}=21.31\times Precipitation+10.82\times Evaporation-11715.31$$ (20)

In the change period (1989~2018), the recharge items of Daihai Lake were precipitation, river water and groundwater, and the discharge items were evaporation and water consumption by Daihai power plant (DPPC). The *Inflow*_*No_Human*_ in change period could be calculated according to the Eq (20). Actual inflow recharge (AIR) = TALE-TALP+ DPPC+VCL. The lake water loss caused by climatic factors (LCF) = TALE -TALP. The lake water loss caused by human activities (LHA) = *Inflow*_*No_Human*_—AIR. Contribution of human impact (CHI) was calculated using the Eq (21).


$$\text{CHI}=\frac{LHA}{LHA+LCF}=\frac{\;Inflow{\;}_{No-Human}-(TALE-TALP+DPPC+VCL)}{\;Inflow_{No-Human}-DPPC-VCL}$$ (21)

The calculation results showed that the CHI was fluctuating upward (Table 10) and 5-year average CHI were 51.33%, 47.46%, 50.21%, 47.19%, 61.99% and 65.62% respectively. In general, the climate factor was the main factor 10 years ago, while the human impact increased rapidly in last 10 years with 5-year average CHI higher than 61.99%. The human factor has been the main factor in the reduction of lake water volume.

**Table 10:** Influence of human activities and climate on water quantity of Daihai Lake.

Year	ACLL	TALP	TALE	DPPC	VCL	AIR	*Inflow*_*No_Human*_	LCF	LHA	CHI
	(m)	(10^4^m^3^)	(10^4^m^3^)	(10^4^m^3^)	(10^4^m^3^)	(10^4^m^3^)	(10^4^m^3^)	(10^4^m^3^)	(10^4^m^3^)	
1989	-0.32	4022.49	9358.79	0	-3191.62	2144.68	7062.05	5336.30	4917.37	47.96%
1990	-0.07	4614.26	8835.50	0	-670.15	3551.09	8271.65	4221.24	4720.56	52.79%
1991	-0.17	4764.34	8251.84	0	-1614.55	1872.95	8111.89	3487.50	6238.93	64.14%
1992	0.04	4446.83	8053.68	0	378.09	3984.94	7512.89	3606.85	3527.95	49.45%
1993	-0.49	2966.41	8489.62	0	-4504.9	1018.30	5004.90	5523.21	3986.60	41.92%
1994	-0.43	2728.38	8544.76	0	-3751.79	2064.60	5447.70	5816.39	3383.10	36.77%
1995	0.52	6148.25	8564.69	0	4558.57	6975.01	14071.77	2416.44	7096.76	74.60%
1996	0.02	4125.92	8403.10	0	180.05	4457.23	8079.27	4277.18	3622.04	45.85%
1997	-0.45	2447.03	9313.51	0	-3970.67	2895.80	5466.23	6866.47	2570.43	27.24%
1998	-0.18	3721.65	8053.49	0	-1530.29	2801.56	7545.33	4331.84	4743.77	52.27%
1999	-0.42	2733.21	7723.30	0	-3480.65	1509.45	5346.70	4990.10	3837.24	43.47%
2000	-0.35	2601.54	7098.10	0	-2804	1692.56	4705.57	4496.56	3013.01	40.12%
2001	-0.47	2491.33	6907.17	0	-3627.73	788.11	4808.28	4415.84	4020.17	47.65%
2002	-0.2	3388.15	5956.87	0	-1510.02	1058.69	6384.31	2568.71	5325.62	67.46%
2003	0.73	4297.45	4613.06	0	5611.85	5927.46	6658.37	315.61	730.91	69.84%
2004	0.22	4139.21	5995.60	0	1765.22	3621.61	7369.81	1856.39	3748.19	66.88%
2005	-0.15	3104.43	6918.73	0	-1205.12	2609.17	5816.13	3814.29	3206.96	45.68%
2006	-0.37	2506.59	7086.42	800	-2902.06	2477.77	4785.84	4579.83	2308.08	33.51%
2007	-0.53	2071.08	6461.32	800	-4011.46	1178.79	3319.91	4390.24	2141.13	32.78%
2008	-0.32	2985.56	5755.78	800	-2342.7	1227.52	5382.34	2770.22	4154.82	60.00%
2009	-0.65	2040.88	6236.68	800	-4599.64	396.16	3935.96	4195.80	3539.80	45.76%
2010	-0.46	3001.16	5228.43	1192	-3142.15	277.11	5900.89	2227.26	5623.78	71.63%
2011	-0.74	1328.29	5410.97	1192	-4851.17	423.51	1481.83	4082.68	1058.32	20.59%
2012	-0.22	3228.53	5045.60	1192	-1407.35	1601.72	7671.88	1817.07	6070.16	76.96%
2013	0	3576.04	5140.15	1192	0	2756.11	9110.59	1564.11	6354.47	80.25%
2014	-0.5	2540.19	4863.48	1192	-3106	409.29	5425.04	2323.29	5015.75	68.34%
2015	-0.61	2205.32	4920.96	1192	-3659.69	247.94	4963.83	2715.63	4715.89	63.46%
2016	-0.52	2255.59	4542.81	1192	-3035.15	444.06	4949.89	2287.22	4505.82	66.33%
2017	-0.61	1883.73	4730.69	1192	-3434.18	604.78	4407.37	2846.96	3802.59	57.19%
2018	-0.16	2507.93	4417.81	800	-864.63	1845.26	6865.09	1909.88	5019.83	72.44%

Note: ACLL: annual change of lake level;

TALP: total amount of lake precipitation;

TALE: total amount of lake evaporation;

DPPC: Daihai power plant consumption;

VCL: volume change of lake;

AIR: actual inflow recharge including surface water and groundwater;

*Inflow*_*No_Human*_: total amount of recharge into the lake including surface water and groundwater with no human disturbance;

LCF: the lake water loss caused by climatic factors;

LHA: the lake water loss caused by human activities;

CHI: contribution of human impact.

In the calculation of CHI, evaporation data was the major source of error which could affect both AIR and Inflow_No-Human_, and TALP, DPPC and VCL were relative accurate values. In present study, potential evaporation was measured by 20 cm-diameter evaporation pan and converted to lake surface evaporation with conversion coefficient of 0.6. The evaporation conversion coefficient of Inner Mongolia range from 0.57 to 0.63 [33], and the higher evaporation conversion coefficient means the higher actual evaporation. CHI with evaporation conversion coefficient of 0.57 and 0.63 were calculated respectively, and the results showed that 5-year average CHI with evaporation conversion coefficient of 0.57 and 0.63 respectively in 1989~2018 were 53.59%, 49.25%, 51.84%, 48.58%, 63.49%, 66.91% and 49.31%, 45.87%, 48.78%, 45.98%, 60.66%, 64.48% respectively (S1 and S2 Tables). The results indicated that the higher lake surface evaporation, the lower CHI. Compared with CHI with evaporation conversion coefficient of 0.6, the error of 5-year average CHI ranged from -2.02% to 2.25% when evaporation conversion coefficient in the range of (0.57, 0.63).

There were some uncertainties in the quantitative calculation of the contribution of climate and human activity. The sources of uncertainty mainly included the selection of the reference period, the climate data of the reference period, and the error of the fitting equation. Firstly, 1960~1970 was selected as the reference period assuming that the inflow into the lake were not disturbed by human activities, while it was hard to meet this assumption as absolute absence of human interference was nearly impossible. In reference period, there were still weak human activities that interfered inflow into the lake and this could affect the final results. Secondly, the precipitation and evaporation data for the reference period were collected from Liangcheng Meteorological Station (near Daihai Town) as reported by Huang [32], and Liangcheng Meteorological Station was about 10 kilometers away from Sansumu Station. Although the distance was not very large, the observed values between these two stations may be still slightly different. Finally, there were many factors affecting the recharge into the lake, and precipitation and evaporation were the most important factors. Using precipitation and evaporation to fit the recharge into the lake according to the regression analysis method will ignore the influence of other factors, and it will also have some impacts on the calculation results. However, these calculations were still meaningful under the condition of insufficient monitoring data. The calculation results could give us some important inspirations and provide directions for further research and policymaking.

### Policy and recommendation

With people’s increasing awareness of environmental protection, people’s expectation of improving the ecological environment is higher than before. Local residents in Daihai has an urgent need to improve the environment of Daihai, and 69% of the local residents thought it was important to improve the environment of Daihai regions [34]. The local government have taken a series of measures to protect Daihai Lake since 2017 including shutting down most of the exploitation wells and closing about 140 km^2^ of irrigated land. Furthermore, Daihai power plant no longer used Daihai Lake water after 2019. These efforts played an important role in reducing the descending speed of Daihai Lake and Daihai Lake water level decreased by -0.18 m in 2018 showing an obvious slower speed compared with previous years. Although these measures have slowed down the decline of the lake level, the lake is still shrinking. Therefore, more effort should be paid to the scientific research of Daihai Lake.

The present study recommended that more attention should be paid on the following aspects: 1) The amount of river water flow into the lake and the groundwater level in the watershed should be monitored continuously; 2) It is necessary to replenish the water from external water source to Daihai Lake in the future.

## Conclusion

Daihai Lake is a typical inland lake in semi-arid region of Northern China and Daihai lake level declines continually in recent decades. There was an obvious mutation point of lake level in 2006, and the descending speed of Daihai Lake accelerated obviously after 2006. The descending speed of Daihai Lake water level post-2006 (-0.46 m/a) was 3.22 times that of pre-2006 (-0.14 m/a). The centroid of Daihai Lake moved 1365.18 m from southwest to northeast during 1989 ~ 2018 with an average speed of 47.08 m/a. A close relationship between groundwater and lake water was found, and groundwater levels around Daihai Lake were affected by irrigation activities noticeably as an obvious downward trend was found during the irrigation period.

The MKT test revealed that the annual evaporation showed a significant downward trend with a rate of approximately -5.33 mm/a, while no significant trend was found in precipitation. Daihai lake water level showed a very weak relationship with evaporation (r = 0.078, p < 0.01) and precipitation (p>0.05) respectively. These results suggested that the reasons for shrinkage of Daihai Lake were complex and the non-climate factor were more and more important.

Daihai Lake was influenced by human activities mainly from LULC, building reservoirs, pumping groundwater and directly consuming Daihai Lake water by DHPP. Different human activity in Daihai wetland affects hydrological circle in different ways, while they all play an important role in the variation of Daihai lake level. It was thought-provoking that DHPP began to consume Daihai lake water in 2006, which was consistent with abrupt change of Daihai lake level. The proportion of human impact was fluctuating upward. Human factors were the main factor of lake water reduction in last 10 years and the 5-year average contribution of human activities to Daihai Lake shrinkage was more than 61.99%. There were some uncertainties in the quantitative calculation of the contribution of climate and human activity including the selection of the reference period, the climate data of the reference period, and the error of the fitting equation. However, these results were still meaningful as they could give us some important inspirations and provide directions for further research and policymaking. To prevent the continuous shrinkage of Daihai Lake, more attention and economic support should be put into the research and management. The present study could provide supports for the local residents using agricultural climate resources and improvement of the ecological environment around the lake area.

## Supporting information

S1 Table Influence of human activities and climate on water quantity of Daihai Lake with evaporation conversion coefficient of 0.57. (DOCX)

S2 Table Influence of human activities and climate on water quantity of Daihai Lake with evaporation conversion coefficient of 0.63. (DOCX)
