# Supplementary material for: Impacts of climate and human activities on Daihai Lake in a typical semi-arid watershed, Northern China
Source: PLoS One. 2022 May 24;17(5):e0266049. doi: 10.1371/journal.pone.0266049 (PMC9129052; doi:10.1371/journal.pone.0266049)
Supplement: S1 Table — (DOCX) [file pone.0266049.s001.docx]

**S1 Table.** **Influence of human activities and climate on water quantity of Daihai Lake with evaporation conversion coefficient of 0.57.**

| **Year** | **ACLL (m)** | **TALP (10^4^m^3^)** | **TALE (10^4^m^3^)** | **DPPC (10^4^m^3^)** | **VCL (10^4^m^3^)** | **AIR (10^4^m^3^)** | **Inflow*_No_Human_* (10^4^m^3^)** | **LCF (10^4^m^3^)** | **LHA (10^4^m^3^)** | **CHI** |
| --- | --- | --- | --- | --- | --- | --- | --- | --- | --- | --- |
|  |  |  |  |  |  |  |  |  |  |  |
| 1989 | -0.32 | 4022.49 | 8890.85 | 0 | -3191.62 | 1676.74 | 6553.55 | 4868.36 | 4876.80 | 50.04% |
| 1990 | -0.07 | 4614.26 | 8393.72 | 0 | -670.15 | 3109.32 | 7778.96 | 3779.47 | 4669.64 | 55.27% |
| 1991 | -0.17 | 4764.34 | 7839.25 | 0 | -1614.55 | 1460.36 | 7647.96 | 3074.91 | 6187.60 | 66.80% |
| 1992 | 0.04 | 4446.83 | 7650.99 | 0 | 378.09 | 3582.26 | 7052.28 | 3204.17 | 3470.02 | 51.99% |
| 1993 | -0.49 | 2966.41 | 8065.14 | 0 | -4504.9 | 593.82 | 4509.65 | 5098.73 | 3915.82 | 43.44% |
| 1994 | -0.43 | 2728.38 | 8117.53 | 0 | -3751.79 | 1637.36 | 4920.82 | 5389.15 | 3283.46 | 37.86% |
| 1995 | 0.52 | 6148.25 | 8136.46 | 0 | 4558.57 | 6546.78 | 13537.56 | 1988.21 | 6990.78 | 77.86% |
| 1996 | 0.02 | 4125.92 | 7982.95 | 0 | 180.05 | 4037.08 | 7576.06 | 3857.03 | 3538.98 | 47.85% |
| 1997 | -0.45 | 2447.03 | 8847.83 | 0 | -3970.67 | 2430.13 | 4900.07 | 6400.80 | 2469.94 | 27.84% |
| 1998 | -0.18 | 3721.65 | 7650.82 | 0 | -1530.29 | 2398.88 | 7041.11 | 3929.17 | 4642.23 | 54.16% |
| 1999 | -0.42 | 2733.21 | 7337.14 | 0 | -3480.65 | 1123.29 | 4843.94 | 4603.93 | 3720.65 | 44.69% |
| 2000 | -0.35 | 2601.54 | 6743.20 | 0 | -2804 | 1337.65 | 4228.69 | 4141.65 | 2891.04 | 41.11% |
| 2001 | -0.47 | 2491.33 | 6561.81 | 0 | -3627.73 | 442.76 | 4325.20 | 4070.48 | 3882.45 | 48.82% |
| 2002 | -0.2 | 3388.15 | 5659.02 | 0 | -1510.02 | 760.85 | 5957.43 | 2270.87 | 5196.58 | 69.59% |
| 2003 | 0.73 | 4297.45 | 4382.41 | 0 | 5611.85 | 5696.81 | 6334.25 | 84.96 | 637.44 | 88.24% |
| 2004 | 0.22 | 4139.21 | 5695.82 | 0 | 1765.22 | 3321.83 | 6965.36 | 1556.61 | 3643.53 | 70.07% |
| 2005 | -0.15 | 3104.43 | 6572.79 | 0 | -1205.12 | 2263.23 | 5350.74 | 3468.36 | 3087.51 | 47.10% |
| 2006 | -0.37 | 2506.59 | 6732.10 | 800 | -2902.06 | 2123.45 | 4299.52 | 4225.51 | 2176.07 | 33.99% |
| 2007 | -0.53 | 2071.08 | 6138.26 | 800 | -4011.46 | 855.72 | 2859.04 | 4067.18 | 2003.32 | 33.00% |
| 2008 | -0.32 | 2985.56 | 5467.99 | 800 | -2342.7 | 939.73 | 4959.42 | 2482.43 | 4019.69 | 61.82% |
| 2009 | -0.65 | 2040.88 | 5924.85 | 800 | -4599.64 | 84.32 | 3460.05 | 3883.96 | 3375.73 | 46.50% |
| 2010 | -0.46 | 3001.16 | 4967.00 | 1192 | -3142.15 | 15.69 | 5487.42 | 1965.84 | 5471.73 | 73.57% |
| 2011 | -0.74 | 1328.29 | 5140.42 | 1192 | -4851.17 | 152.96 | 1037.00 | 3812.13 | 884.03 | 18.82% |
| 2012 | -0.22 | 3228.53 | 4793.32 | 1192 | -1407.35 | 1349.44 | 7242.95 | 1564.79 | 5893.52 | 79.02% |
| 2013 | 0 | 3576.04 | 4883.15 | 1192 | 0 | 2499.11 | 8671.21 | 1307.11 | 6172.10 | 82.52% |
| 2014 | -0.5 | 2540.19 | 4620.31 | 1192 | -3106 | 166.12 | 5002.55 | 2080.12 | 4836.43 | 69.93% |
| 2015 | -0.61 | 2205.32 | 4674.91 | 1192 | -3659.69 | 1.89 | 4520.82 | 2469.58 | 4518.93 | 64.66% |
| 2016 | -0.52 | 2255.59 | 4315.67 | 1192 | -3035.15 | 216.92 | 4528.56 | 2060.08 | 4311.63 | 67.67% |
| 2017 | -0.61 | 1883.73 | 4494.16 | 1192 | -3434.18 | 368.24 | 3955.52 | 2610.42 | 3587.28 | 57.88% |
| 2018 | -0.16 | 2507.93 | 4196.92 | 800 | -864.63 | 1624.37 | 6426.42 | 1688.99 | 4802.06 | 73.98% |

Note: ACLL: annual change of lake level;

TALP: total amount of lake pricipation;

TALE: total amount of lake evaporation;

DPPC: Daihai power plant consumption;

VCL: volume change of lake;

AIR: actual inflow recharge including surface water and groundwater;

*Inflow_No_Human_*: total amount of recharge into the lake including surface water and groundwater with no human disturbance;

LCF: the lake water loss caused by climatic factors;

LHA: the lake water loss caused by human activities;

CHI: contribution of human impact.
