# Supplementary material for: Impacts of climate and human activities on Daihai Lake in a typical semi-arid watershed, Northern China
Source: PLoS One. 2022 May 24;17(5):e0266049. doi: 10.1371/journal.pone.0266049 (PMC9129052; doi:10.1371/journal.pone.0266049)
Supplement: S2 Table — (DOCX) [file pone.0266049.s002.docx]

**S2 Table. Influence of human activities and climate on water quantity of Daihai Lake with evaporation conversion coefficient of 0.63.**

| **Year** | **ACLL (m)** | **TALP (10^4^m^3^)** | **TALE (10^4^m^3^)** | **DPPC (10^4^m^3^)** | **VCL (10^4^m^3^)** | **AIR (10^4^m^3^)** | **Inflow*_No_Human_* (10^4^m^3^)** | **LCF (10^4^m^3^)** | **LHA (10^4^m^3^)** | **CHI** |
| --- | --- | --- | --- | --- | --- | --- | --- | --- | --- | --- |
|  |  |  |  |  |  |  |  |  |  |  |
| 1989 | -0.32 | 4022.49 | 9826.73 | 0 | -3191.62 | 2612.62 | 7570.55 | 5804.24 | 4957.93 | 46.07% |
| 1990 | -0.07 | 4614.26 | 9277.27 | 0 | -670.15 | 3992.87 | 8764.34 | 4663.02 | 4771.47 | 50.57% |
| 1991 | -0.17 | 4764.34 | 8664.43 | 0 | -1614.55 | 2285.54 | 8575.81 | 3900.09 | 6290.27 | 61.73% |
| 1992 | 0.04 | 4446.83 | 8456.36 | 0 | 378.09 | 4387.62 | 7973.50 | 4009.53 | 3585.88 | 47.21% |
| 1993 | -0.49 | 2966.41 | 8914.10 | 0 | -4504.9 | 1442.79 | 5500.16 | 5947.69 | 4057.37 | 40.55% |
| 1994 | -0.43 | 2728.38 | 8972.00 | 0 | -3751.79 | 2491.84 | 5974.58 | 6243.62 | 3482.74 | 35.81% |
| 1995 | 0.52 | 6148.25 | 8992.93 | 0 | 4558.57 | 7403.25 | 14605.99 | 2844.68 | 7202.74 | 71.69% |
| 1996 | 0.02 | 4125.92 | 8823.26 | 0 | 180.05 | 4877.39 | 8582.48 | 4697.34 | 3705.09 | 44.10% |
| 1997 | -0.45 | 2447.03 | 9779.18 | 0 | -3970.67 | 3361.48 | 6032.40 | 7332.15 | 2670.92 | 26.70% |
| 1998 | -0.18 | 3721.65 | 8456.17 | 0 | -1530.29 | 3204.23 | 8049.54 | 4734.52 | 4845.31 | 50.58% |
| 1999 | -0.42 | 2733.21 | 8109.47 | 0 | -3480.65 | 1895.62 | 5849.45 | 5376.26 | 3953.83 | 42.38% |
| 2000 | -0.35 | 2601.54 | 7453.01 | 0 | -2804 | 2047.46 | 5182.45 | 4851.46 | 3134.98 | 39.25% |
| 2001 | -0.47 | 2491.33 | 7252.53 | 0 | -3627.73 | 1133.47 | 5291.36 | 4761.20 | 4157.89 | 46.62% |
| 2002 | -0.2 | 3388.15 | 6254.71 | 0 | -1510.02 | 1356.53 | 6811.19 | 2866.56 | 5454.65 | 65.55% |
| 2003 | 0.73 | 4297.45 | 4843.71 | 0 | 5611.85 | 6158.11 | 6982.49 | 546.26 | 824.37 | 60.15% |
| 2004 | 0.22 | 4139.21 | 6295.38 | 0 | 1765.22 | 3921.39 | 7774.25 | 2156.17 | 3852.86 | 64.12% |
| 2005 | -0.15 | 3104.43 | 7264.66 | 0 | -1205.12 | 2955.11 | 6281.52 | 4160.23 | 3326.41 | 44.43% |
| 2006 | -0.37 | 2506.59 | 7440.74 | 800 | -2902.06 | 2832.09 | 5272.17 | 4934.15 | 2440.08 | 33.09% |
| 2007 | -0.53 | 2071.08 | 6784.39 | 800 | -4011.46 | 1501.85 | 3780.79 | 4713.31 | 2278.94 | 32.59% |
| 2008 | -0.32 | 2985.56 | 6043.57 | 800 | -2342.7 | 1515.31 | 5805.26 | 3058.01 | 4289.95 | 58.38% |
| 2009 | -0.65 | 2040.88 | 6548.52 | 800 | -4599.64 | 707.99 | 4411.86 | 4507.63 | 3703.87 | 45.11% |
| 2010 | -0.46 | 3001.16 | 5489.85 | 1192 | -3142.15 | 538.53 | 6314.36 | 2488.69 | 5775.83 | 69.89% |
| 2011 | -0.74 | 1328.29 | 5681.52 | 1192 | -4851.17 | 694.06 | 1926.66 | 4353.23 | 1232.60 | 22.07% |
| 2012 | -0.22 | 3228.53 | 5297.88 | 1192 | -1407.35 | 1854.00 | 8100.80 | 2069.35 | 6246.81 | 75.12% |
| 2013 | 0 | 3576.04 | 5397.16 | 1192 | 0 | 3013.12 | 9549.96 | 1821.12 | 6536.84 | 78.21% |
| 2014 | -0.5 | 2540.19 | 5106.66 | 1192 | -3106 | 652.47 | 5847.54 | 2566.47 | 5195.07 | 66.93% |
| 2015 | -0.61 | 2205.32 | 5167.00 | 1192 | -3659.69 | 493.99 | 5406.85 | 2961.68 | 4912.86 | 62.39% |
| 2016 | -0.52 | 2255.59 | 4769.95 | 1192 | -3035.15 | 671.20 | 5371.21 | 2514.36 | 4700.01 | 65.15% |
| 2017 | -0.61 | 1883.73 | 4967.23 | 1192 | -3434.18 | 841.31 | 4859.22 | 3083.49 | 4017.91 | 56.58% |
| 2018 | -0.16 | 2507.93 | 4638.70 | 800 | -864.63 | 2066.15 | 7303.75 | 2130.78 | 5237.61 | 71.08% |

Note: ACLL: annual change of lake level;

TALP: total amount of lake pricipation;

TALE: total amount of lake evaporation;

DPPC: Daihai power plant consumption;

VCL: volume change of lake;

AIR: actual inflow recharge including surface water and groundwater;

*Inflow_No_Human_*: total amount of recharge into the lake including surface water and groundwater with no human disturbance

LCF: the lake water loss caused by climatic factors;

LHA: the lake water loss caused by human activities;

CHI: contribution of human impact.
